# Association between the systemic inflammation response index and serum uric acid in acute traumatic brain injury: a cross-sectional study

**DOI:** 10.3389/fneur.2026.1803502

**Published:** 2026-05-12

**Authors:** Jinrong Wang, Hong Huang, Keyu Dai, Zicong Li, Liguo Zhang, Hua Liu

**Affiliations:** 1 Department of Neurosurgery, Affiliated Kunshan Hospital of Jiangsu University, Kunshan, Jiangsu, China; 2 School of Medicine, Nanjing Medical University, Nanjing, Jiangsu, China; 3 Department of Neurosurgery, Shibei Hospital of Jing’an District, Shanghai, China; 4 Department of Neurosurgery, Northern Jiangsu People’s Hospital, The Yangzhou School of Clinical Medicine of Dalian Medical University, Yangzhou, China

**Keywords:** immunometabolic coupling, inflammation, systemic inflammation response index, traumatic brain injury, uric acid

## Abstract

**Background:**

Traumatic brain injury (TBI) involves systemic inflammation, oxidative stress, and metabolic disturbances. The systemic inflammation response index (SIRI) and serum uric acid (UA) are both linked to inflammatory and oxidative processes, but their relationship in acute TBI is unclear.

**Objective:**

To examine the association between SIRI and serum UA in adults with acute TBI, including potential nonlinear patterns and differences by sex and injury severity.

**Methods:**

This retrospective cross-sectional study included 1,930 adults with CT-confirmed acute TBI admitted between March 2018 and April 2024. Laboratory data were collected within 24 h of admission. SIRI was calculated as neutrophil × monocyte/lymphocyte. Multivariable linear regression, generalized additive models, and piecewise linear regression were used to assess the association between SIRI and UA. Subgroup analyses were conducted by sex and Glasgow Coma Scale (GCS) category.

**Results:**

Higher SIRI was independently associated with higher UA levels. In the fully adjusted model, each 1-unit increase in SIRI corresponded to a 0.88 μmol/L increase in UA (*β* = 0.88, 95% CI: 0.29–1.47). The relationship was approximately linear, and the threshold model did not significantly improve fit. The association was significant in both sexes, stronger in females, and significant only in patients with severe TBI (GCS 3–8).

**Conclusion:**

Higher SIRI was associated with increased serum UA in acute TBI, suggesting a link between systemic inflammation and purine metabolism after injury. This relationship may be stronger in females and in severe TBI. Further longitudinal multicenter studies are needed.

## Introduction

Traumatic brain injury (TBI) remains a major cause of mortality and long-term disability worldwide. In addition to the initial mechanical insult, secondary injury processes—including systemic inflammatory activation, oxidative stress, and metabolic dysregulation—play critical roles in determining clinical outcomes (1–6). Increasing evidence suggests that TBI is not merely a focal neurological disorder, but rather a systemic condition characterized by complex interactions between the central nervous system and peripheral immune responses (2–5).

Among circulating biomarkers, the systemic inflammation response index (SIRI), which integrates neutrophil, monocyte, and lymphocyte counts, has emerged as a comprehensive indicator of systemic inflammatory status. Compared with single leukocyte-based parameters, SIRI may more effectively reflect the balance between innate and adaptive immune responses and has demonstrated prognostic value in a variety of acute and critical illnesses, including TBI (7).

Serum uric acid (UA), the final product of purine metabolism, is closely associated with oxidative stress and inflammatory pathways (8, 9). However, its role in TBI remains controversial, with some studies suggesting antioxidant and potentially protective effects, whereas others have reported deleterious pro-inflammatory associations (1, 6). Despite growing interest in both inflammatory biomarkers and metabolic alterations in TBI, these two domains have largely been investigated separately (1, 6, 10–12).

From a pathophysiological perspective, systemic inflammation may influence purine metabolism through several mechanisms, including increased cellular turnover, activation of xanthine oxidase pathways, and oxidative stress–related metabolic shifts (8, 9). This raises the possibility that inflammatory activation and uric acid metabolism are interconnected processes during the acute phase of TBI, reflecting a broader phenomenon of “immunometabolic coupling.”

Importantly, TBI represents a unique clinical context in which neuroinflammation, blood–brain barrier disruption, and neuroendocrine stress responses interact with systemic immune activation (2–5). These features may lead to distinct immunometabolic alterations that differ from those observed in other acute inflammatory or traumatic conditions.

Therefore, we hypothesized that systemic inflammation, as reflected by SIRI, is associated with alterations in serum uric acid levels in patients with acute TBI. To test this hypothesis, we conducted a large retrospective study to evaluate the association between SIRI and UA, explore potential nonlinear relationships, and assess whether this association varied according to sex and injury severity.

## Methods

### Study design and setting

This cross-sectional study was conducted at Kunshan Hospital, affiliated with Jiangsu University. Adult patients admitted with acute traumatic brain injury between March 2018 and April 2024 were consecutively screened for eligibility. The study protocol was approved by the Ethics Committee of Kunshan Hospital (Approval No. 2022–06-025) and was conducted in accordance with the Declaration of Helsinki.

Given the retrospective nature of the study and the use of routinely collected anonymized clinical data, the requirement for written informed consent was waived by the Ethics Committee. All identifiable patient information was removed before analysis to ensure confidentiality.

### Participants

Eligible participants were adults aged 18 years or older with acute TBI confirmed by computed tomography (CT) and complete relevant laboratory data obtained within 24 h of hospital admission. Patients were excluded if they had missing key variables (including laboratory results, body mass index, or Glasgow Coma Scale score), non-traumatic causes of brain injury such as stroke, a history of malignancy, early inter-hospital transfer, or a hospital stay of less than 1 day. A total of 1,930 patients were included in the final analysis.

### Exposure and outcome

The primary exposure of interest was the systemic inflammation response index (SIRI), calculated from complete blood count parameters using the following formula:


SIRI=(neutrophil count×monocyte count)/lymphocyte count

All parameters were expressed in the same unit (10^9^/L). In the statistical analyses, SIRI was examined both as a continuous variable and as a categorical variable based on quartiles.

The primary outcome was serum uric acid (UA), expressed in μmol/L and measured using standard clinical laboratory methods.

### Covariates

Potential confounders were selected *a priori* on the basis of clinical relevance and previous literature. The multivariable models adjusted for demographic factors (age and sex), body mass index (BMI), laboratory parameters [alanine aminotransferase (ALT), hemoglobin (Hb), and estimated glomerular filtration rate (eGFR)], comorbidities (diabetes and hypertension), neurological status assessed by the Glasgow Coma Scale (GCS), and the presence of concomitant extracranial injuries.

### Statistical analysis

Continuous variables are presented as means with standard deviations (SDs), and categorical variables as frequencies and percentages. Baseline characteristics were compared across quartiles of SIRI, defined according to the 25th, 50th, and 75th percentiles of the sample distribution.

The association between SIRI and serum UA was assessed using multivariable linear regression models with stepwise adjustment. Model 1 was unadjusted. Model 2 was adjusted for age, sex, and BMI. Model 3 was fully adjusted for ALT, Hb, eGFR, diabetes, hypertension, GCS score, and concomitant injuries.

Potential nonlinearity was evaluated using generalized additive models (GAMs). Smoothing was performed using spline-based functions with automatically selected smoothing parameters based on the data, as implemented in EmpowerStats (R-based). Piecewise linear regression was further applied to explore potential threshold effects, and model fit was compared using the likelihood ratio test.

Extreme values of continuous variables were handled by winsorization at the 1st and 99th percentiles to minimize the influence of outliers. Multicollinearity among covariates was assessed using variance inflation factor (VIF) values, with VIF > 5 considered indicative of potential collinearity.

A complete-case analysis was used to handle missing data. Patients with missing values for any variable included in the models were excluded, accounting for approximately 25% of the initial cohort.

Subgroup analyses were performed stratified by sex and GCS categories (mild: 13–15; moderate: 9–12; severe: 3–8). All statistical tests were two-sided, with a significance level of *α* = 0.05. Analyses were conducted using EmpowerStats and R software.

## Results

### Baseline characteristics

Among the 1,930 patients included in the analysis, higher SIRI quartiles were associated with younger age and a greater proportion of male patients (both *p* < 0.001). ALT and UA increased across SIRI quartiles, whereas Hb decreased (all *p* < 0.001). The proportion of severe TBI (GCS 3–8) increased markedly from 40.58% in Q1 to 71.22% in Q4 (*p* < 0.001), as did the prevalence of concomitant injuries (7.25% vs. 17.39%, p < 0.001). BMI and eGFR were comparable across quartiles, whereas diabetes differed significantly (*p* = 0.021).

### Univariate associations with UA

In univariate analyses, UA was positively associated with BMI, ALT, Hb, and SIRI, and negatively associated with age and eGFR. Female sex was associated with lower UA levels compared with male sex. Moderate TBI severity (GCS 9–12 vs. 13–15) was associated with lower UA, whereas severe TBI was not significantly associated with UA in the unadjusted analysis.

### Multivariable association between SIRI and UA

SIRI was consistently associated with higher UA levels across all models. In the unadjusted model, each 1-unit increase in SIRI corresponded to a 1.81 μmol/L increase in UA (*β* = 1.81, 95% CI: 1.17–2.44). After adjustment for age, sex, and BMI, the association was attenuated but remained statistically significant (*β* = 1.21, 95% CI: 0.62–1.80). In the fully adjusted model, SIRI remained independently associated with UA (*β* = 0.88, 95% CI: 0.29–1.47).

### Nonlinearity and threshold effect

**Figure 1:**
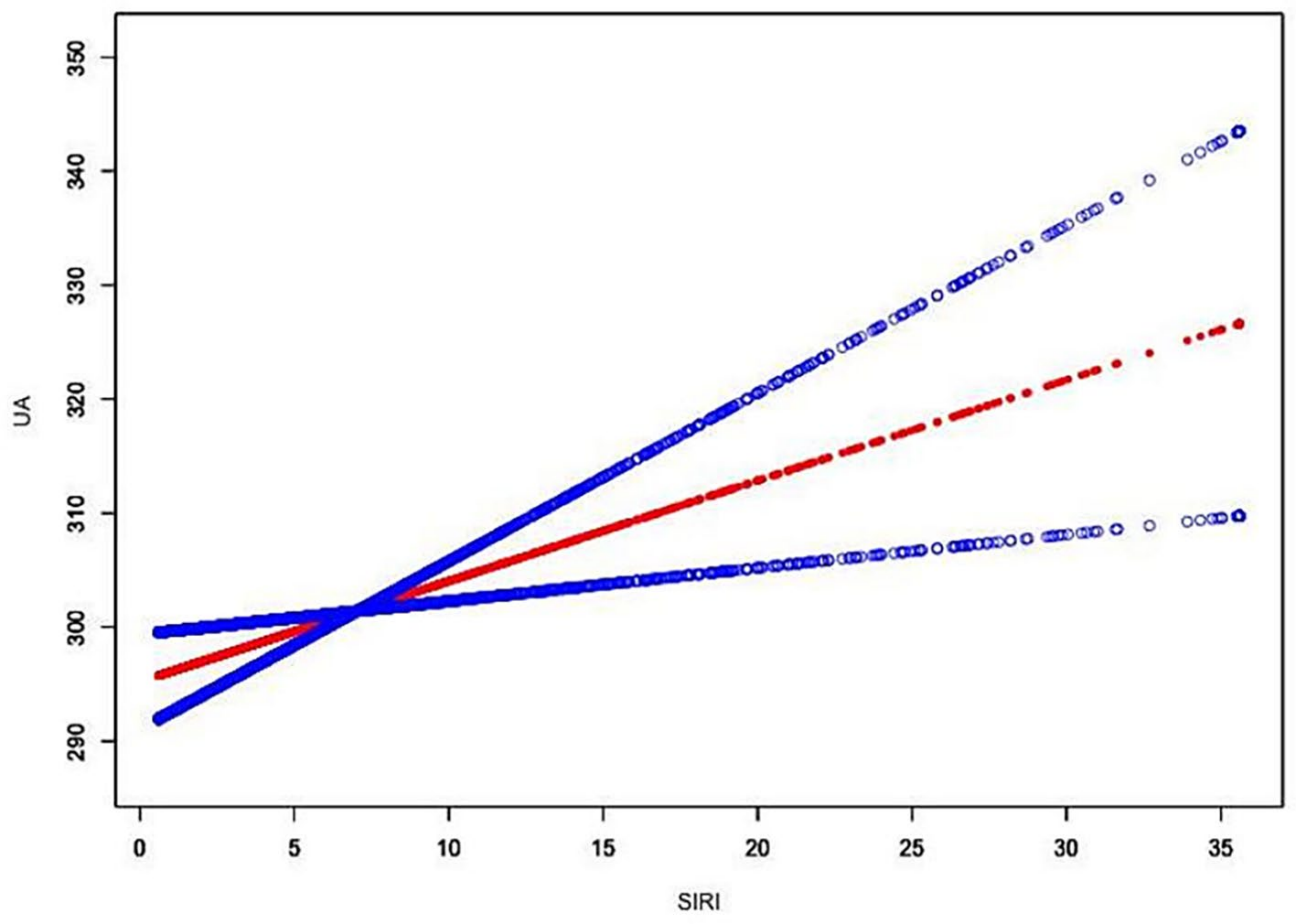
Adjusted smooth curve of the association between SIRI and serum uric acid (UA) in patients with traumatic brain injury (GAM).

The GAM-based smoothing curve suggested an approximately linear positive association between SIRI and UA (see Figure 1). Piecewise regression identified a potential inflection point at SIRI = 1.05. Below this point, the association was not statistically significant, whereas above this point, the association was significant (*β* = 0.95, 95% CI: 0.35–1.55). However, the likelihood ratio test did not support the threshold model over the linear model (*p* = 0.154), indicating that the overall relationship was better characterized as linear.

### Subgroup analyses

In sex-stratified analyses, SIRI remained positively associated with UA in both males (*β* = 0.75, 95% CI: 0.06–1.44) and females (*β* = 1.51, 95% CI: 0.35–2.67).

In GCS-stratified analyses, a statistically significant association was observed only among patients with severe TBI (GCS 3–8; *β* = 1.02, 95% CI: 0.28–1.76), whereas the associations in mild and moderate TBI were not statistically significant (see Tables 1–5).

**Table 1:** Baseline characteristics of patients stratified by SIRI quartiles.

Variable	Q1 (*n* = 483)	Q2 (*n* = 482)	Q3 (*n* = 482)	Q4 (*n* = 483)	*p*	*p* for trend
BMI, kg/m^2^	23.68 ± 3.84	23.63 ± 3.46	23.54 ± 3.63	23.41 ± 3.32	0.663	0.792
Age, years	53.42 ± 16.59	52.25 ± 16.47	50.21 ± 16.29	49.67 ± 16.72	<0.001	0.001
ALT, U/L	23.54 ± 21.34	26.49 ± 20.35	31.39 ± 44.76	35.50 ± 39.07	<0.001	<0.001
eGFR, mL/min/1.73 m^2^	109.79 ± 19.50	111.17 ± 20.49	111.30 ± 21.14	111.80 ± 20.21	0.458	0.137
Uric acid, μmol/L	287.29 ± 96.04	297.87 ± 94.76	304.78 ± 95.64	315.72 ± 108.49	<0.001	<0.001
Hemoglobin, g/L	129.38 ± 20.11	128.39 ± 24.29	128.61 ± 26.62	119.44 ± 36.20	<0.001	<0.001
Sex					<0.001	NA
Male	286 (59.21%)	327 (67.84%)	378 (78.42%)	379 (78.47%)		
Female	197 (40.79%)	155 (32.16%)	104 (21.58%)	104 (21.53%)		
Other injury					<0.001	NA
No	448 (92.75%)	445 (92.32%)	414 (85.89%)	399 (82.61%)		
Yes	35 (7.25%)	37 (7.68%)	68 (14.11%)	84 (17.39%)		
Diabetes					0.021	NA
No	454 (94.00%)	428 (88.80%)	445 (92.32%)	448 (92.75%)		
Yes	29 (6.00%)	54 (11.20%)	37 (7.68%)	35 (7.25%)		
Hypertension					0.928	NA
No	308 (63.77%)	298 (61.83%)	306 (63.49%)	305 (63.15%)		
Yes	175 (36.23%)	184 (38.17%)	176 (36.51%)	178 (36.85%)		
GCS category					<0.001	NA
Mild (13–15)	142 (29.40%)	128 (26.56%)	94 (19.50%)	70 (14.49%)		
Moderate (9–12)	145 (30.02%)	120 (24.90%)	106 (21.99%)	69 (14.29%)		
Severe (3–8)	196 (40.58%)	234 (48.55%)	282 (58.51%)	344 (71.22%)		

Data are presented as mean ± standard deviation (SD) for continuous variables and n (%) for categorical variables. *p*-values were calculated using the Kruskal–Wallis test for continuous variables and the chi-square test or Fisher’s exact test, as appropriate, for categorical variables. *p* for trend was calculated across SIRI quartiles for continuous variables.

**Table 2:** Univariable linear regression analysis of factors associated with uric acid.

Variable	Mean ± SD or *n* (%)	*β* (95% CI)	*p*
Age, years	51.39 ± 16.58	−0.90 (−1.17, −0.64)	<0.0001
Sex			
Male	1,370 (70.98%)	Reference	
Female	560 (29.02%)	−72.42 (−81.64, −63.20)	<0.0001
BMI, kg/m^2^	23.57 ± 3.56	7.22 (6.02, 8.42)	<0.0001
ALT, U/L	29.23 ± 33.45	0.70 (0.57, 0.83)	<0.0001
Hemoglobin, g/L	126.45 ± 27.73	0.57 (0.41, 0.73)	<0.0001
eGFR, mL/min/1.73 m^2^	111.01 ± 20.34	−0.87 (−1.09, −0.66)	<0.0001
Diabetes			
No	1775 (91.97%)	Reference	
Yes	155 (8.03%)	−1.89 (−18.21, 14.42)	0.8203
Hypertension			
No	1,217 (63.06%)	Reference	
Yes	713 (36.94%)	7.50 (−1.68, 16.68)	0.1095
GCS category			
Mild (13–15)	434 (22.49%)	Reference	
Moderate (9–12)	440 (22.80%)	−16.55 (−29.71, −3.39)	0.0138
Severe (3–8)	1,056 (54.72%)	−8.78 (−19.87, 2.31)	0.1209
Other injury			
No	1706 (88.39%)	Reference	
Yes	224 (11.61%)	−3.60 (−17.44, 10.24)	0.6102
SIRI	7.03 ± 6.95	1.81 (1.17, 2.44)	<0.0001

Data are presented as mean ± standard deviation (SD) for continuous variables and *n* (%) for categorical variables. *β* represents the unstandardized regression coefficient with 95% confidence interval (CI) obtained from univariable linear regression models with uric acid as the dependent variable. Reference indicates the reference category for categorical variables. All statistical tests were two-sided, and *p* < 0.05 was considered statistically significant. BMI, body mass index; ALT, alanine aminotransferase; eGFR, estimated glomerular filtration rate; GCS, Glasgow Coma Scale; SIRI, systemic inflammatory response index.

**Table 3:** Threshold effect analysis of the association between SIRI and uric acid.

Model	Effect	*β* (95% CI)	*p* value
Model I	Linear effect	0.88 (0.29, 1.47)	0.0035
Model II	Threshold (K)	1.05	
	Slope 1 (< K)	−38.90 (−93.92, 16.13)	0.1661
	Slope 2 (> K)	0.95 (0.35, 1.55)	0.0019
	Difference in slope (Slope 2 vs. Slope 1)	39.85 (−15.27, 94.96)	0.1567
	Predicted UA at threshold	290.42 (284.39, 296.46)	
	Likelihood ratio test	0.154	

*β* represents the unstandardized regression coefficient with 95% confidence interval (CI) for the association between SIRI and uric acid. Model I assumes a linear association between SIRI and uric acid. Model II is a two-piecewise linear regression model with an inflection point (K) identified from the data. Slope 1 and Slope 2 represent the effect estimates below and above the threshold, respectively. The likelihood ratio test compares Model II with Model I; a *p* < 0.05 indicates that the segmented model provides a significantly better fit than the linear model. All tests were two-sided. SIRI, systemic inflammatory response index; UA, uric acid.

**Table 4:** Association between SIRI and serum uric acid in linear regression models (*n* = 1,930).

Exposure	Model 0 (Crude), *β* (95% CI)	*p*	Model 1, *β* (95% CI)	*p*	Model 2, *β* (95% CI)	*p*
SIRI	1.81 (1.17, 2.44)	<0.01	1.21 (0.62, 1.80)	<0.01	0.88 (0.29, 1.47)	<0.01

UA (μmol/L) was treated as the dependent variable. *β* represents the mean change in UA (μmol/L) associated with each 1-unit increase in SIRI. Model 0 was unadjusted. Model 1 was adjusted for age, sex, and body mass index (BMI). Model 2 was further adjusted for alanine aminotransferase (ALT), hemoglobin, estimated glomerular filtration rate (eGFR), diabetes, hypertension, Glasgow Coma Scale (GCS) category, and other injury. All *p*-values were two-sided. SIRI, systemic inflammatory response index; UA, uric acid; CI, confidence interval; BMI, body mass index; ALT, alanine aminotransferase; eGFR, estimated glomerular filtration rate; GCS, Glasgow Coma Scale.

**Table 5:** Stratified analyses of the association between SIRI and uric acid using adjusted linear regression models.

Stratification variable	Subgroup	*n*	*β* (95% CI)	*p*
Sex	Male	1,370	0.75 (0.06, 1.44)	0.0322
	Female	560	1.51 (0.35, 2.67)	0.0113
GCS category	Mild (13–15)	434	0.85 (−0.50, 2.20)	0.2159
	Moderate (9–12)	440	0.89 (−0.48, 2.25)	0.2032
	Severe (3–8)	1,056	1.02 (0.28, 1.76)	0.0073

UA was treated as the dependent variable. *β* represents the mean change in UA associated with each 1-unit increase in SIRI. In sex-stratified analyses, models were adjusted for age, body mass index (BMI), alanine aminotransferase (ALT), hemoglobin, estimated glomerular filtration rate (eGFR), diabetes, hypertension, GCS category, and other injury. In GCS-stratified analyses, models were adjusted for age, sex, body mass index (BMI), alanine aminotransferase (ALT), hemoglobin, estimated glomerular filtration rate (eGFR), diabetes, hypertension, and other injury. SIRI, systemic inflammatory response index; UA, uric acid; CI, confidence interval; BMI, body mass index; ALT, alanine aminotransferase; eGFR, estimated glomerular filtration rate; GCS, Glasgow Coma Scale.

## Discussion

In this large cross-sectional study of 1,930 patients with acute traumatic brain injury, we found that a higher systemic inflammation response index (SIRI) was independently associated with elevated serum uric acid levels after adjustment for multiple confounders. These findings support a link between systemic inflammatory activation and purine metabolism in the acute phase of TBI, in line with the concept of immunometabolic coupling (1, 7–9).

This association is biologically plausible in the setting of acute TBI. Following brain injury, a cascade of secondary events is triggered, including neuroinflammation, blood–brain barrier disruption, glial activation, and peripheral immune responses (2–5, 13). TBI has increasingly been recognized as a systemic disorder rather than an isolated cerebral insult, with neuroimmune interactions extending beyond the central nervous system (6, 14). Experimental and clinical studies further suggest that neuroendocrine-immune dysfunction and brain–gut axis disturbances may contribute to broader systemic inflammatory and metabolic consequences after injury (15–17).

Within this context, several mechanisms may explain the observed relationship between SIRI and UA. Acute tissue injury and inflammatory activation can promote cellular turnover and oxidative stress, thereby enhancing purine catabolism and uric acid generation (8, 9, 18). At the same time, inflammatory signaling pathways, including inflammasome-related responses, are increasingly implicated in post-traumatic immune dysregulation (19–22). Uric acid itself may also play a dual role: under some conditions it functions as an antioxidant, whereas in others it may amplify inflammatory signaling and metabolic injury (6, 21, 23). This bidirectional interaction supports the notion that inflammation and uric acid metabolism are closely intertwined during the acute phase of TBI.

Recent work has also emphasized the importance of metabolic adaptation and host–microbiome interactions after brain injury. Changes in gut microbiota composition and microbiota-related metabolites have been linked to neuroinflammation and systemic immune responses following TBI (17, 24–26). More broadly, intestinal microecology has been implicated in the development of hyperuricemia and inflammatory metabolic disorders (24, 27). In parallel, experimental studies indicate that post-TBI metabolic remodeling involves coordinated changes in inflammatory and cellular energetic pathways (28, 29). Together, these findings provide further support for an immunometabolic framework through which systemic inflammation may be associated with altered uric acid homeostasis after TBI (30).

Notably, the association between SIRI and UA appeared to be stronger in female patients. This finding may reflect sex-related differences in immune and metabolic responses. Females are known to exhibit distinct inflammatory and vascular responses after brain injury, potentially influenced by sex hormones that modulate oxidative stress and immune activation (31). In addition, baseline uric acid levels and renal urate handling differ by sex (32), and inflammasome-related mechanisms may also contribute to sex-specific metabolic heterogeneity (21). These factors may partly explain why the relationship between systemic inflammation and uric acid metabolism was more pronounced in female patients.

Another important finding was that the association was statistically significant only in patients with severe TBI (39, 40). This may be explained by the more pronounced neuroinflammatory and systemic stress responses associated with severe injury. Patients with severe TBI usually experience greater tissue destruction, more extensive blood–brain barrier dysfunction, and stronger activation of glial, immune, and neuroendocrine pathways (3, 4, 16). Such changes are likely to intensify both systemic inflammatory burden and downstream metabolic disturbances, making the association between SIRI and UA more readily detectable in this subgroup (6, 33). By contrast, in mild or moderate TBI, the degree of systemic perturbation may be insufficient to produce measurable changes in uric acid levels.

From a clinical perspective, our findings support the potential value of integrating inflammatory and metabolic biomarkers in the assessment of acute TBI. SIRI is easily derived from routine blood counts, and prior studies have shown that it has prognostic significance in TBI (7) as well as in other acute inflammatory conditions (34, 35). Similar observations have been reported for related composite inflammatory indices in pediatric TBI (36). At the same time, blood-based biomarkers are increasingly recognized as useful tools for evaluating injury severity and prognosis in TBI (10–12, 37). Against this background, the observed association between SIRI and UA suggests that combined immunometabolic profiling may help identify patients with more pronounced systemic dysregulation, particularly in severe cases (1).

Our study has several strengths. The relatively large sample size enhanced statistical power, and the use of multivariable regression models with comprehensive adjustment reduced the likelihood of confounding. In addition, the application of generalized additive models enabled us to assess potential nonlinear relationships and supported the robustness of the observed linear association.

However, several limitations should be acknowledged. First, owing to the cross-sectional design, causal relationships cannot be established. Second, this was a single-center study involving a relatively homogeneous population, which may limit the generalizability of the findings. Third, although multiple covariates were adjusted for, residual confounding cannot be excluded. In addition, the study relied on routinely collected clinical data and lacked more advanced experimental methods, limiting mechanistic insight. Finally, the absence of longitudinal data precluded assessment of dynamic changes over time.

Future studies should focus on prospective validation in multicenter cohorts and further elucidate the biological mechanisms underlying the interaction between systemic inflammation and uric acid metabolism. Longitudinal investigations incorporating repeated measurements and mechanistic approaches may help clarify temporal relationships and improve clinical applicability (6, 28, 29, 38).

## Limitations

Several limitations should be acknowledged. First, due to the cross-sectional design, causal relationships cannot be established. Second, this was a single-center study with a relatively homogeneous population, which may limit the generalizability of the findings; multi-center studies with larger and more diverse populations are needed. Third, although multiple covariates were adjusted for, residual confounding cannot be excluded given the limited scope of available data. Finally, the lack of longitudinal measurements prevented assessment of dynamic changes over time. Future studies with repeated measurements and more advanced approaches may provide deeper insights into the underlying mechanisms.

## Conclusion

In adults with acute traumatic brain injury, higher systemic inflammation response index levels were independently associated with elevated serum uric acid concentrations. These findings indicate an association, rather than a causal relationship, between systemic inflammatory activity and purine metabolism in the acute phase of TBI. This association appeared to be more pronounced in female patients and in those with severe injury, suggesting heterogeneity in immunometabolic responses following brain injury. Further longitudinal and multicenter studies are warranted to clarify the temporal nature and clinical significance of these findings.

## Data Availability

The original contributions presented in the study are included in the article/supplementary material, further inquiries can be directed to the corresponding author.
